# Parisian Correspondence

**Published:** 1854-04

**Authors:** 


### *Parisian Correspondence, the opening of the College Courses, the Academy of Medicine, the perchloride of Iron treatment for Anuerisms, treatment of Cholera.*

A number of American Physicians have visited Paris, France, during the past year, from whom letters have been received and published and generally highly interesting. They describe graphically, and some of them, minutely, the opening of the courses for the session, and give a programme of the order of the various departments.

The faculty consists of Denonvilliers, Berard, Moquin, Tandon, Wartz, Garvarret, Dumeril, Reguin, Gerdy, Cloquet, Andral, Grisolle, Velpeau, Dubois, with many others, whose names are familiar to the American profession, and who have made themselves illustrious by their numerous and valuable contributions to medical science.

On the occasion of the opening, all the Professors were fantastically enrobed in gowns of red silk, a mantle of fur over the shoulder, a cap upon the head, with the trappings and flummeries of official consequence. Paul Dubois was Dean, and proclaimed the opening of the "seance," and also that M. Bouchardot would speak, who proceeded to pronounce a eulogy upon Royal-Collard and also upon Richards, late professors respectively of Hygiene and Botany.

The profession of our country was most ably represented by Professors Brainard, Judkins, Hall and others. The former has made himself conspicuous by some valuable contributions, the other, a learned gentleman, a close observer, and distinguished anatomist, was able to detect many egregious blunders in their diagnosis, and the last has watched with care and industry, the practice in the different Hospitals and by different men, and has made a most able report of his observations. To these men and all such this parade, pomp, and empty show, must have proved disgusting, because trifling.

A stated meeting of the Academy of Medicine was held, in the beginning of which, the scene is described as being "confusion worse confounded," and to have resembled more a riotous political assemblage than that of a body of men of so dignified a character as to be deserving badges of distinction and marks declarative of their superiorty, but a short time previous in another place. The jabbering in French "*lingo*," with their rapid gesticulations and their quaint expressions of countenance, together with every variety of tone in their voices, from the deep sepulchral hollow note, to the fine stridulous tone, made it a scene rather painfully interesting and ludicrous in the extreme. After continued efforts on the part of the President, order was at last established.

The use of the Perchloride of Iron in Anuerisms was then discussed. From M. Malaign's report it would appear that the bright hopes of M. Pravaz, its discoverer, were not fulfilled, justified, nor confirmed. He hoped to immortalize himself by this discovery as valuable in medical science as lithotrity for stone. The modus operandi is to introduce a fine canula into the tumor, then inject the perchloride, to the amount of four drops into the current of the blood as it flows within the tumor.

A warm discussion followed, however, between the most prominent members, among whom were M. Malgaigne, Valpeau, Gerdy, Moreau and others. The discussion was continued. The cholera was prevailing there at the time, and, from the letters written on the subject, we cull the following as the treatment which is pursued by the distinguished men of Paris, for that disease.

M. Aran changes his treatment to suit the case before him. He uses alternately alcoholic and aromatic drinks with opium and nitrate silver and astringent injections. He tried the saline treatment, the solution of muriate soda dissolved in gum water by injections. He uses mustard baths and gives bi-carb soda in drinks. He thinks the saline treatment justifies further, and a more extended trial. The indication in the use of this is based in part upon the physiological fact that the salts in the blood are the vehicles and instruments of vitalizing it by oxygenation. He also gives juleps with creosote and laudanum; of the former he gives to the amount of 15 drops—to 25 of the latter. He has used Iodine in combination with laudanum. Ice with wine and alcohol is used by Guerard. Rostan adds the vapor bath. Grisolle adds blisters to the epigastrium.

In examinations after death it was found that the blood was much more fluid than in any other disease, that there is albumen in the urine, and that the muciferous glands in the colon and rectum were enlarged and Peyers plates thickened and ulcerated.

In the La Charite cases were prescribed for by Rayer, Cruvelhier, Andral, and others of prominence. Rayer uses wet cupping on the epigastrium, on the walls of the abdomen and at the same time he prescribes opiates. He never cups where there is cyanosis or when the pulse bespeaks a failure of the vital forces, uses stimulants in the cold stage, warmth internal and external.

Cruveilhier uses stimulating drinks, sinapisms, and blisters, the latter moved from place to place, Andral punch and sinapisms. Piory open windows, fresh air, and albumen in drinks, Brignet laudanum in drinks and in injections.

Such then is the treatment of the principal men of these Hospitals, in which there is not a grain of calomel, acetate of lead, or strycnia, or turpentine or any other remedy of much power, except in the narcotics as used by Brignet, the creosote and nitras argent by Aran. For our part it does appear to us as next to no treatment, which indeed may be as successful as any other. We would prefer to take Brignet's course, if compelled to choose from among them.

Dr. Brainard, of Chicago, read a paper before the "Society of Surgery," on the infiltration of Iodine in Spina Bifida, Hydrocepalus Œdema, Erysipelas, &c. We are indebted to the New York Medical Gazette for the facts contained in the above in relation to the mode of treating Cholera in a letter by that Journal's correspondent.

Versatility is a characteristic trait in the French nation. This is more manifest in their political, social and civil relations than in their literary and scientific pursuits, and yet these last have not been entirely free from this peculiarity. But considering the mutability of their government, their revolutions and counter revolutions, their intestine feuds and bloody struggles, they have certainly exhibited a devotion to, and perseverance in, the cultivation of science, highly commendable to its votaries and worthy the example and imitation of other civilized nations, which have been less racked by civil convulsions. The secret of their success under these trying circumstances, is doubtless owing to the fact that whatever may be the form of government and whoever may be in authority, the "powers that be" always have thrown their ægis over those engaged in scientific enquiry, and liberally endowed their institutions. The people too, *en masse*, with singular unanimity and liberality, always bestowed their smiles upon those who were devoting their lives to research, and almost deified those who had arisen to prominence and fame. The patriot and warrior always secured from that people the liberal tribute of glory and gratitude, but the man of science always wore a brighter green laurel and a more dazzling brilliant in his coronet. Where there is such a premium offered to those who excel, and such inducements constantly held out, they are encouraged to an energetic prosecution of their enquiries, and an untiring research into the undiscovered truths of science, and cannot fail, but will persevere until they will acquire that elevated position in the world of science to which they are destined to attain.

The tendency of the votaries and disciples of medicine there, is to the adoption of materialism in their systems and yet with this fault they will develop such substantial facts and truths as will yet shed a bright light upon hitherto doubtful questions. Their animal chemistry, their delicate microscopic researches and investigations, their pathological enquiries, and their anatomical minutia, are now fountains from which flow streams full and flowing, irrigating and vitalizing the great field of science and will render it productive, in a high degree, of fruitful and fortunate results to humanity.

But why this tide of American medical men to France? Why neglect our old ancestral kinsmen, from whom we have descended and from whom we have derived our own full, beautiful, and expressive language. Hunter, Abernethy, Watson, Hall and the Coopers, to say nothing of numerous other bright luminaries, were enough of themselves to make medical science immortal. What a flood of bright and enduring light they have reflected upon its pathway. How profound their erudition and how patient the research, and still not more patient than *careful*. They do not jump at conclusions, but when an opinion is formed and promulged, it is done upon the most indubitable testimony. Slow and careful in their progress, they are therefore less under the necessity to change, and if they do not *appear* to advance, they nevertheless make as rapid progress as their neighbors. After all we admire them as men of deep science and although we dislike John Bull in politics, we admire John Bull's science. Their works carry with them the weight of authority, and will continue to be monuments of wisdom in coming time and will be regarded as evidences of talent and erudition as some of the choice ancient works are still regarded. Why then, we repeat, not visit London? Do they exercise toward us, whom they regard as their recusant descendants, an intolerant spirit and look coldly and inhospitably toward those who may aspire to a high rank in science, and jealous of this, discourage an approach within their temples and their halls? It would appear that this is the true state of things, as well as a reluctance to aknowledge merit elsewhere than in their own little circumscribed isle, which leads them to entertain feelings of jealousy toward all others, and especially when they see that ere long the proud position they have ever occupied for solid literature and science, will soon be equalled by those who have descended from her, but whom, in moments of national unkindness, she attempted to subdue and coerce. We have derived however, substantial benefit from British literature and science, though doled out to us with that miserly reluctance which savored more of compulsion than a free and neighborly act. Let us have a Medical Congress of all enlightened nations, break down these conventionalisms, and make our intercourse to be free and unrestrained. In this, foreign States will realize as much benefit to their departments of literature and science, as will ourselves, for the American medical profession is now a full grown power and can reflex as much benefit to others as she will derive from them. We are comparatively independent, and if a non-intercourse be established with our English neighbors, we have abundant resources of our own. But as a nation we are not selfish. We welcome our neighbors among us and the only fault we commit is in an excess of attention. We do not wrap ourselves in robes of self consciousness and with an air of superlative assumption say, "we are holier than thou," as is charged to their account.

The attention paid to Marshall Hall, was not only deservedly bestowed, but showed triumphantly that our countrymen entertain no spirit of envy, but will continue to award to distingushed merit that mead it deserves, whatever may be the rule of *their* conduct. The American medical profession indulge in no complaint nor do they murmur because of advantages denied. By no means. Medical literature has derived as much, if not more, from the founts of science in this country, as those of any other nation in proportion to the time of our national existence; and for every favor we have received, since then, we have made full and ample return. No, no, we only propose an exchange and an interchange, in order that both may derive benefit, as there is mental development by the attrition of mind with mind.

We had prepared some remarks touching the standing and notions of *some* who go abroad and their conduct after their return, but we will not apply the *sinapism* now.

